# Isolation and biochemical characterization of a metagenome-derived 3-deoxy-d-arabino-heptulosonate-7-phosphate synthase gene from subtropical marine mangrove wetland sediments

**DOI:** 10.1186/s13568-019-0742-4

**Published:** 2019-02-04

**Authors:** Huaxian Zhao, Hua Gao, Kai Ji, Bing Yan, Quanwen Li, Shuming Mo, Minggang Zheng, Qian Ou, Bo Wu, Nan Li, Chengjian Jiang

**Affiliations:** 1 State Key Laboratory for Conservation and Utilization of Subtropical Agro-bioresources, College of Life Science and Technology, Guangxi University, 100 Daxue East Road, Nanning, 530004 Guangxi People’s Republic of China; 2 Guangxi Key Laboratory of Mangrove Conservation and Utilization, Guangxi Mangrove Research Center, Guangxi Academy of Sciences, 92 Changqing Road, Beihai, 536000 Guangxi People’s Republic of China; 3 Key Laboratory of Environment Change and Resources Use in Beibu Gulf (Guangxi Teachers Education University), Ministry of Education, 175 Mingxiu East Road, Nanning, 530001 Guangxi People’s Republic of China; 4 The First Institute of Oceanography, State Oceanic Administration of China, 6 XianXiaLing Road, Qingdao, 266061 People’s Republic of China

**Keywords:** 3-Deoxy-d-arabino-heptulosonate-7-phosphate synthase, Novel gene, Metagenomic library, Subtropical marine mangrove sediment, Biochemical characterization

## Abstract

3-Deoxy-d-arabino-heptulosonate-7-phosphate synthase (DAHPS) is a key rate-limiting enzyme in aromatic amino acid anabolism. A new I_β_-type DAHPS gene (*aro1A*) was identified in a metagenomic library from subtropical marine mangrove sediment. The gene encoded a polypeptide composed of 272 amino acids and had a maximum similarity of 52.4% to a known DAHPS at the amino acid level. Multiple sequence alignment, homologous modeling, and molecular docking showed that Aro1A had the typical (β/α)_8_ barrel-shaped catalytic structural domain of DAHPS. The motifs and amino acid residues involved in the combination of substrates and metal ligand were highly conservative with the known DAHPS. The putative DAHPS gene was subcloned into a pET-30a(+) vector and was overexpressed in *Escherichia coli* Rosetta (DE3) cells. The recombinant protein was purified to homogeneity. The maximum activity for the recombinant Aro1A protein occurred at pH 8.0 and 40 °C. Ba^2+^ and Ca^2+^ stimulated the activity of Aro1A protein. The enzyme showed high affinity and catalytic efficiency (*K*_*m*_^PEP^ = 19.58 μM, *V*_*max*_^PEP^  = 29.02 μM min^−1^, and *k*_cat_^PEP^/*K*_*m*_^PEP^ = 0.88 s^−1^ μM^−1^) under optimal reaction conditions. The enzymatic property of Aro1A indicates its potential in aromatic amino acid industrial production.

**Electronic supplementary material:**

The online version of this article (10.1186/s13568-019-0742-4) contains supplementary material, which is available to authorized users.

## Introduction

3-Deoxy-d-arabino-heptulosonate-7-phosphate synthase (DAHPS) is a key rate-limiting enzyme in the synthesis of aromatic amino acids, such as phenylalanine, tyrosine, and tryptophan (Herrmann 1995). This enzyme can catalyze phosphoenolpyruvate (PEP), d-erythrose-4-phosphate (E4P), and H_2_O to form 3-deoxy-d-arabino-heptulosonate-7-phosphate (DAHP) in the first step of shikimic acid approach. Shikimic acid approach mainly exists in bacteria, fungi, and vegetation but not inside higher animals (Herrmann 1995). Therefore, DAHPS from pathogenic microorganisms, including *Mycobacterium tuberculosis* and *Neisseria meningitidis*, becomes the antibacterial target candidate (Cross et al. 2013; Webby et al. 2005a). Studies on the engineered strains aim to produce shikimic acid or aromatic amino acid (like L-Phe) to relieve the feedback inhibition of DAHPS and improve the yield (Cui et al. 2014; Liu et al. 2013). Thus, DAHPS received research interest for medical field and industrial production.

DAHPSs can be classified as type I or type II according to their molecular dimension: type I is < 40 kDa, and type II is 50 kDa (Gosset et al. 2001; Jensen et al. 2002). Type I DAHPS is divided into types I_α_ and I_β_ (Jensen et al. 2002). The DAHPSs from *Escherichia coli*, *Saccharomyces cerevisiae*, and *N. meningitidis* represent type I_α_. The N terminus of DAHPS has a regulating region that inhibits enzymatic activity by combining with Phe, Tyr, and Trp. Type I_β_ DAHPS is divided into two types. The first type includes a feedback regulation domain, whereas the other type does not have a feedback regulation domain. The recently discovered regulation domains include chorismate mutase and ferredoxin-like domains (Table 1). Among these domains, the most common is chorismate mutase located in the N terminus, such as the DAHPSs from *Bacillus subtilis* and *Listeria monocytogenes* (Light et al. 2012; Pratap et al. 2017). The feedback inhibition of various type I_β_ DAHPSs is more complicated than that of type I_α_ DAHPS. Type I_β_ DAHPS is inhibited by downstream aromatic amino acids, including Phe, Tyr, Trp, chorismate, and prephenate, in independent or cooperative ways. Type I_β_ DAHPS without a regulation domain is not generally inhibited by downstream aromatic amino acids (Table 1). Meanwhile, type II DAHPS includes DAHPSs from vegetation and certain microorganisms such as *M. tuberculosis, Corynebacterium glutamicum*, and *Helicobacter pylori.* These representatives are inhibited similarly as types I_α_ and I_β_ DAHPS. Types I and II DAHPSs have no apparent sequence similarity (Shumilin et al. 2004). The polymer form also varied among different DAHPSs. Recent research shows that the activity of the DAHPS from *Providencia alcalifaciens* is affected by its oligomeric state (Sharma et al. 2018). However, all the reported DAHPSs have similar (β/α)_8_ barrel-shaped catalytic structural domain, and their catalytic activity depends on a divalent metal ion (Wu et al. 2005).

**Table 1:** Representative DAHPSs from different types of microorganisms

Protein	Organism	T	Feedback structure	Feedback inhibitor	PF	References
AroG, AroF, AroH	*Escherichia coli*	I_α_	N-Region	Phe, Tyr, Trp	2, 4	Ray and Bauerle (1991); Schoner and Herrmann (1976); Shumilin et al. (1999, 2004)
Aro3, Aro4	*Saccharomyces cerevisiae*	I_α_	N-Region	Phe, Tyr, Trp	2, 4, 8	Helmstaedt et al. (2005); König et al. (2004); Künzler et al. (1992); Teshiba et al. (1986)
*Nme* DS	*Neisseria meningitidis*	I_α_	N-Region	Phe, Tyr, Trp	4	Cross et al. (2013); Heyes et al. (2014)
DAHP^Pae^	*Pseudomonas aeruginosa*	I_α_	–	–	–	Sterritt et al. (2018)
*Tm* DS	*Thermotoga maritima*	I_β_	N-Ferredoxin-like domain	Phe, Tyr	2, 4	Shumilin et al. (2004); Wu et al. (2003)
*Pfu* DS	*Pyrococcus furiosus*	I_β_	None	None of Phe, Tyr, and Trp	2, 4	Schofield et al. (2004, 2005)
*Gsp* DS	*Geobacillus* sp.	I_β_	N-Chorismate mutase	Prephenate	4	Nazmi et al. (2016)
*Ap* DS	*Aeropyrum pernix*	I_β_	None	None of Phe, Tyr, Trp, chorismate, shikimate, and prephenate,	4	Zhou et al. (2012)
aroA(Q)^168^	*Bacillus subtilis*	I_β_	N-Chorismate mutase	Chorismate, Prephenate	4	Wu et al. (2005)
*Pg* DS	*Porphyromonas gingivalis*	I_β_	C-Chorismate mutase	Chorismate, prephenate		Wu and Woodard (2006)
*Lm* DS	*Listeria monocytogenes*	I_β_	N-Chorismate mutase	Chorismate, prephenate	4	Light et al. (2012)
*Pae* DAHPS^PA2843^	*Pseudomonas aeruginosa*	II	N-Region	Trp	4	Sterritt et al. (2018)
*Mt* DAHPS	*Mycobacterium tuberculosis*	II	N-Chorismate mutase	Phe/Tyr, Phe/Trp, Tyr/Trp, Tyr, Trp, and chorismate	2, 4	Light et al. (2012); Webby et al. (2010)
AroG, AroF, AroH	*Corynebacterium glutamicum*	II	N-Region	Phe, Tyr, Trp	2, 4	Burschowsky et al. (2018); Liu et al. (2008)
*Hpy* DS	*Helicobacter pylori*	II	Unknown	None of Phe, Tyr, Trp, and chorismate	2	Webby et al. (2005b)

*T* DAHPS type, *PF* polymer form

To date, more than 99% microorganisms cannot be cultivated under pure-cultured conditions (Amann et al. 1995). Metagenomic technology that is not cultivation-dependent was developed to overcome limitations in studying genes that come from microorganisms that cannot be cultivated (Amann et al. 1995). Metagenome-derived amylases, cellulases, esterases, polyketide synthases, and alkaline proteases were identified using function-based and sequence-based screening strategies (Leis et al. 2015; Mewis et al. 2013; Niehaus et al. 2011; Seow et al. 1997; Yang et al. 2016; Yun et al. 2004). Most of these enzymes have new physio-biochemical characteristics and provide rich research materials for the improvement of industrial enzymes and for the further investigation of enzyme structures and functions.

Herein, a plasmid metagenomic library was constructed successfully from subtropical marine mangrove wetland sediments by using pUC118 as the cloning vector. A new gene (*aro1A*) encoding DAHPS was cloned and identified. To our knowledge, this gene is the first metagenome-derived DAHPS from subtropical marine mangrove sediment. The gene provided new materials and theoretical references for the industrial production of aromatic amino acids.

## Materials and methods

### Strains and plasmids

The host strain of the metagenomic library was *E. coli* DH5α (Novagen), which was also used to construct and preserve recombinant expression plasmids. *E. coli* Rosetta (DE3) (Novagen) was used for the expression of recombinant proteins. Plasmid pUC118 *Hin*cII/BAP (Takara) was the vector carrying the metagenomic library, and plasmid pET-30a(+) (Novagen) was the expression vector.

### Construction of the metagenomic library

A sample of 0–10 cm-deep sediment was collected from a mangrove surrounding the intertidal zone in Beihai City, Guangxi Province, China (N21°26′28″, E109°11′37″). The sediment sample had a temperature of 30 °C and a pH of 5.5. A high-quality metagenomic DNA was extracted from the sample by using a FastDNA SPIN kit (MP Biomedicals, USA) according to the manufacturer’s protocols (Additional file 1: Fig. S1A). The inserted DNA was the 2–6 kb gel-extracted fragments from the mixture of equal amounts of products digested with *Hin*cII and *Sma*I. This inserted DNA was ligated to the pUC118 *Hin*cII/BAP (Takara), and 5 μL of ligation products were transformed into 50 μL of electro-competent *E. coli* DH5α. The transformed cells were recovered using 1 mL of SOC medium at 37 °C and 180 rpm. The same batches of recovery culture were combined. To calculate the size of the DNA fragments in the library, we placed 5 μL of cultured samples in LB agar plates containing 100 μg mL^−1^ of ampicillin, 40 μg mL^−1^ of 5-bromo-4-chloro-3-indolyl-β-d-galactopyranoside, and 40 μg mL^−1^ of isopropyl β-d-1-thiogalactopyranoside (IPTG). The combined culture was allowed to grow for 3 h, and then 5 μL of the cultured sample was collected to calculate the proliferation fold. The rest of the cells were stored in 20% glycerinum at − 80 °C after centrifugation. The library was stored at − 80 °C until screening.

### Isolation and sequence analysis of *aro1A*

DNA sequence analysis was performed using a BigDye terminator cycle sequencing kit on an ABI Prism 3700 DNA analyzer (Applied Biosystems, USA). Open reading frame (ORF) analysis of all clone inserts was performed using the ORF Finder (NCBI). ORF annotation was based on the results of blastx and CD-Search from NCBI. The target gene *aro1A* in this study was derived from a positive clone (pUME11) and was annotated as a DAHPS gene based on sequence analysis.

The molecular weight and the theoretical isoelectric point of the protein were predicted via ProtParam (Gasteiger et al. 2005). Furthermore, sequence identification and conserved domain analysis of the protein were performed using the BLAST and CD-Search programs from NCBI, respectively (Marchler-Bauer et al. 2017). Phylogenetic analysis was performed using the MEGA7 software (Kumar et al. 2016). The evolutionary history was inferred using the neighbor-joining method. Multiple sequence alignment was performed via the Clustal OMEGA program (Sievers et al. 2014), and the alignment result was slightly adjusted to align the conserved sites, according to previous studies. The secondary structure information revealed in the alignment was obtained from the 3D structure data of the protein. The predicted structure of Aro1A was built automatically using the SWISS-MODEL server (Waterhouse et al. 2018).

### Combination patterns of Aro1A and ligands

The combination patterns of Aro1A and ligands were predicted via the AutoDock 4.2.6 program (Morris et al. 2009). The receptor was the predicted structure of Aro1A. The ligands PEP and E4P were obtained from the DAHPS structures of *Aeropyrum pernix* (PDB: 1VS1) and *Thermotoga maritima* (PDB: 1RZM), respectively. The atoms of the receptor were assigned to “AD4 type.” The “Grid box” was set to maximum, and the “Search Parameter” was the “genetic algorithm.” The “Number of GA Runs” was set to 200, and default values were used for the remaining parameters.

### Overexpression and purification of the recombinant DAHPS protein

The plasmid containing *aro1A* was extracted as the template for the polymerase chain reaction (PCR). The forward primer (5′-CGGAAGCTTGCATGATGGCCCCATTGGTAACACAAA-3′) and the reverse primer (5′-GGACTCGAGCACCAACTCCCTGTCTATAGCTGCC-3′) were designed based on the nucleotide sequence of *aro1A*, and the restriction enzyme sites for *Hin*dIII and *Xho*I were underlined in the above-mentioned primers, respectively. PCR was performed in a 50 μL reactor consisting of 1× PrimeSTAR buffer (Takara), 1.25 U PrimeSTAR HS DNA polymerase (Takara), 4 μL of dNTP mixture (2.5 mM) (Takara), 0.2 μM forward primer, 0.2 μM reverse primer, 50 ng plasmid, and H_2_O. The PCR program was as follows: 30 cycles at 98 °C for 10 s and at 68 °C for 60 s. The PCR product (Additional file 1: Fig. S2) was purified after being digested with *Hin*dIII and *Xho*I at 37 °C for 3 h. The purified product was ligated to the *Hin*dIII and *Xho*I double-digested vector pET-30a(+) with T4 ligase (Takara). The recombinant plasmid pET-30a(+)-*aro1A* was confirmed by double digestion with *Hin*dIII and *Xho*I (Additional file 1: Fig. S3) and was sequenced by Sangon Biotech (Shanghai). The corresponding recombinant plasmid was transformed into competent *E. coli* Rosetta (DE3) cells. The clone obtained via double-enzyme digestion and sequencing was used for the recombinant protein expression.

A single colony of the protein expression strain *E. coli* Rosetta (DE3)/pET30a(+)-*aro1A* was inoculated into 10 mL of LB-kanamycin (50 µg mL^−1^) and was allowed to grow for 8 h at 37 °C. Then, 3 mL of culture was added to 200 mL of LB-kanamycin (50 µg mL^−1^) containing 0.5 M sorbitol in a 500 mL flask. The resulting mixture was agitated (180 rpm) at 37 °C. IPTG was added to the final concentration of 0.1 mM when the OD_600_ was 0.4–0.6, and the culture was agitated (180 rpm) for 8 h at 16 °C. The His-tagged Aro1A protein was purified from the sonicated lysate of harvested cells by using His60 Ni Superflow Resin (Takara) according to the manufacturer’s instructions. The protein concentration was analyzed using the BCA Protein Assay Kit (Solarbio, China). The expression of the protein was detected and analyzed using SDS–PAGE.

### Assay of Aro1A activity

The assay method for Aro1A was modified as previously described (Nazmi et al. 2016). The reaction mixture solution (1 mL) was composed of phosphate buffer saline (pH 6.8, 10 mM), PEP (25 μM), E4P (25 μM), and CoCl_2_ (0.1 mM). The reaction mixture was incubated at 25 °C for 5 min, and the reaction was initiated by adding Aro1A protein (2 μg). The activity of Aro1A was examined by monitoring the PEP consumption at 232 nm. One unit of enzyme was defined as the amount of Aro1A that converts 1 μmol PEP in 1 min at pH 6.8 and 25 °C.

### Effect of temperature, pH, and divalent metal ion on enzyme activity

The optimal reaction time was studied at 25 °C and pH 6.8. The enzymatic reaction progress was monitored by the change in OD_232_ in the reaction system, in which the initial substrate concentration was 0.25 μM. The optimum reaction time of 10 min was observed based on the reaction progress curve (Additional file 1: Fig. S4).

Temperature-dependent assays were performed at 4 °C–55 °C and pH 6.8 for 10 min, and those that are pH dependent were performed at pH 4.0–9.0 and at the optimum reaction temperature for 10 min. To determine thermostability, we incubated the enzyme at 4 °C–55 °C for 2 h. The assays were performed at optimal reaction conditions. To draw the relative enzyme activity curve of the assays above, we measured the highest enzyme activity in each assay at 100%. Furthermore, to determine the activation of Aro1A, we measured the different divalent metal ions in different reactions, each containing 5 mM metal ion, at optimal reaction conditions by using the standard assay (0.1 mM CoCl_2_) as the control (100%).

### Kinetic data

Reaction velocity was measured when 0.1–0.5 μM PEP was used as the substrate under optimal conditions. The *K*_*m*_^PEP^, *V*_*max*_^PEP^, and *k*_cat_^PEP^ of Aro1A were calculated from the Lineweaver–Burk plot. All reactions were performed in three independent experiments.

### Nucleotide sequence accession number

The *aro1A* nucleotide sequence was deposited in the GenBank database with the accession number MH757446.

## Results

### Construction of metagenomic library and isolation of *aro1A* gene

Two blunt endonucleases of *Hin*cII and *Sma*I for the metagenomic DNA preparation (Additional file 1: Fig. S1B) were used to obtain diverse fragments. The 2–6 kb DNA fragments from the mixture of equal amounts of enzyme-digested products were ligated to linear blunt-end plasmid pUC118 *Hin*cII/BAP. Through blue-white screening, 15 white clones were randomly selected to verify the insertion size (Additional file 1: Fig. S1C). The constructed metagenomic library contained approximately 750,000 clones, the average insertion size was roughly 4 kb, and the metagenomes covered approximately 3.0 Gb. The target gene in this study was from a clone named pUME11 and was annotated as a DAHPS gene based on the sequence analysis. The gene was named *aro1A*, which was 819 bp long.

### Phylogenic relationship and primary structure of Aro1A

**Fig. 1:**
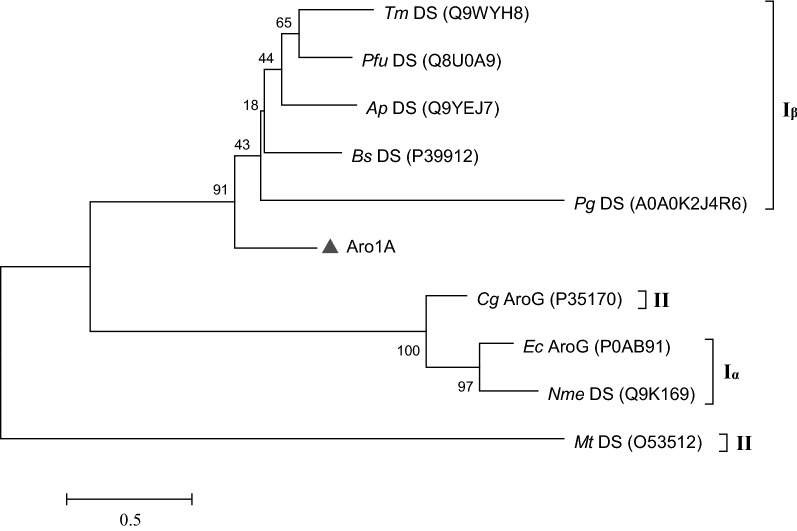
Phylogenetic tree of Aro1A and other DAHPSs. These proteins came from *T. maritima* (*Tm* DS), *P. furiosus* (*Pfu* DS), *A. pernix* (*Ap* DS), *B. subtilis* (*Bs* DS), *P. gingivalis* (*Pg* DS), *C. glutamicum* (*Cg* AroG), *E. coli* (*Ec* AroG), *N. meningitidis* (*Nme* DS), and *M. tuberculosis* (*Mt* DS). The percentage of replicate trees in which the associated replicates were clustered together in the bootstrap test (1000 replicates) is shown next to the branches. Protein accession numbers are in the parentheses

An estimate based on bioinformatics analysis indicated that Aro1A encoded a polypeptide composed of 272 amino acids and had a theoretical isoelectric point of 4.76 and a theoretical molecular weight of 28.82 kDa. The conserved domain analysis tool, CD-Search of NCBI, annotated that Aro1A was a new member of type I DAHPS super family. Aro1A had the highest similarity of 52.4% to the DAHPS from *T. maritima* MSB8 (Accession number: Q9WYH8). Phylogenetic analysis showed that the evolutionary relationship of Aro1A with type I_β_ DAHPS was higher than that with type II or type I_α_ DAHPS (Fig. 1).

**Fig. 2:**
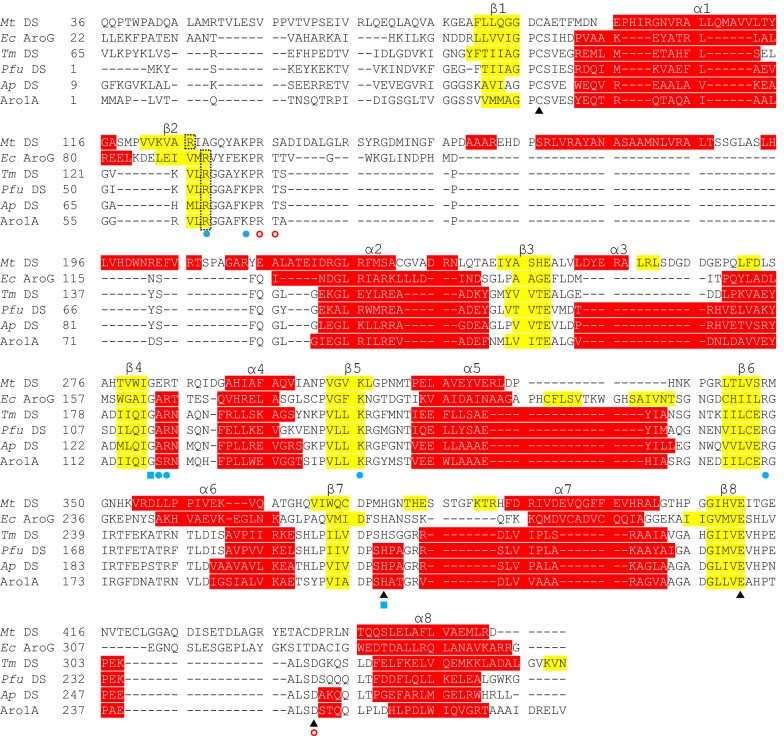
Sequence alignment based on the structure of Aro1A and the five representative DAHPSs. These proteins came from *M. tuberculosis* (*Mt* DS), *E. coli* (*Ec* AroG), *T. maritima* (*Tm* DS), *P. furiosus* (*Pfu* DS), and *A. pernix* (*Ap* DS). The α-helices are highlighted in red, and the β-strands are highlighted in yellow. Conserved divalent metal binding sites are indicated with “black triangle”; conserved PEP binding sites are indicated with “blue circle”; conserved PEP non-bonded contact sites are indicated with “blue square”; and conserved E4P binding sites are indicated with “empty circle”

DAHPS enzymes from *M. tuberculosis* (PDB: 2B7O), *E. coli (*PDB: 1QR7), *T. maritima* (PDB: 1RZM), *Pyrococcus furiosus* (PDB: 4C1K), and *A. pernix* (PDB: 1VS1) were selected as the representative sequences of types I_α_, I_β_, and II, which were multiple-aligned with the Aro1A protein. Multiple sequence alignment results revealed that Aro1A and the other DAHPSs shared similar motif sites (Fig. 2). The divalent metal binding sites of C36, H206, E232, and D243 of Aro1A were consistent with those of the representative DAHPS. The conserved residues of R60, K65, S119, R120, K141, and R171 in Aro1A protein were annotated as the PEP binding sites; G118 and H206 were possibly the conserved amino acid residues in the substrate-binding motif, which had non-bond contact with PEP; R67, T68, and D243 were the possible binding sites of E4P. Figure 2 shows that the properties of secondary structure (e.g. length and amino acid residues) of Aro1A was slightly different from the other DAHPSs, especially *Mt* DAHPS.

### Molecular model and substrate docking analysis of Aro1A

**Fig. 3:**
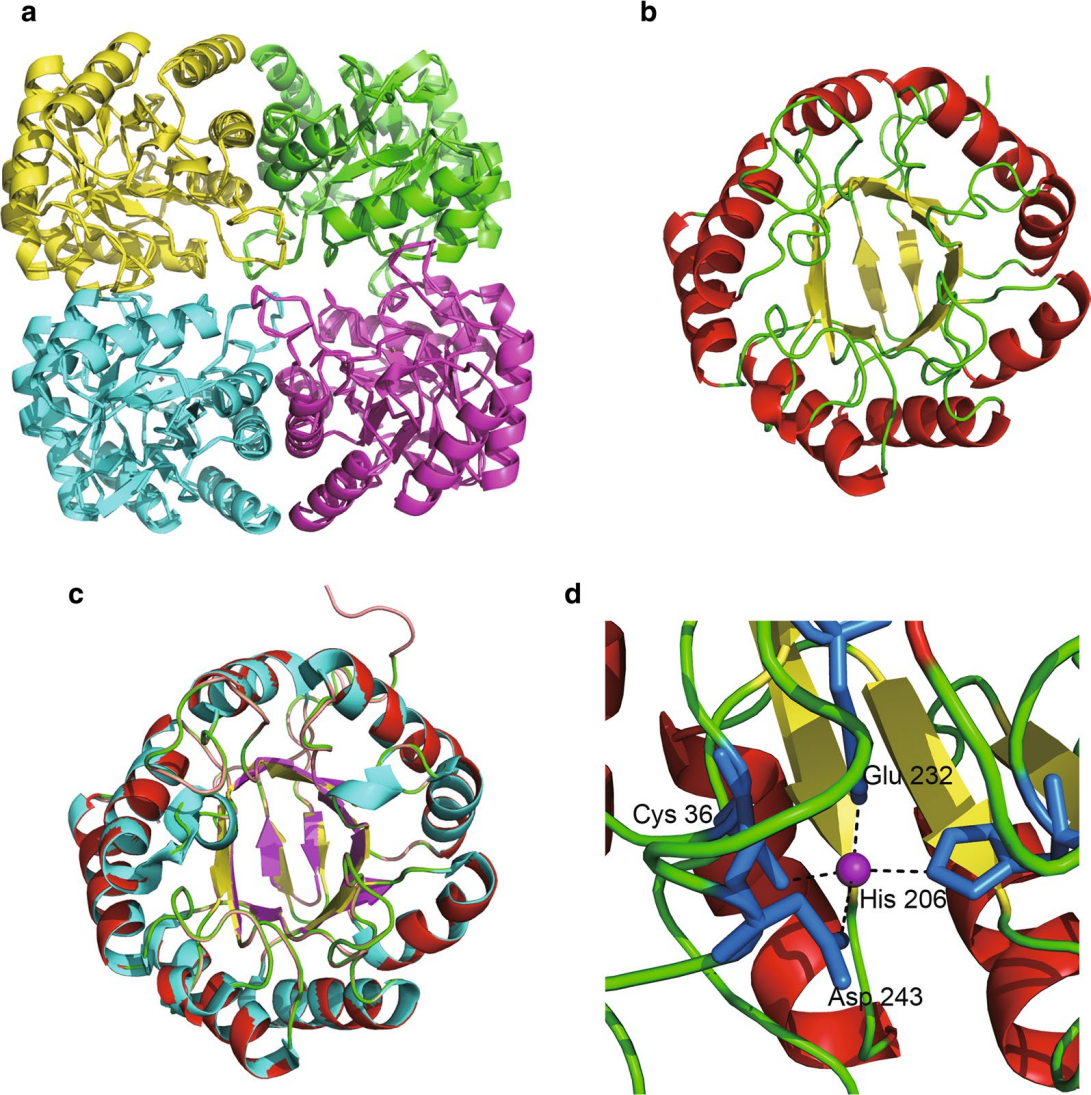
Predicting structure and ligand interaction sites of Aro1A. **a** Tetrameric form of Aro1A. **b** Monomeric form of Aro1A. **c** Superposition of Aro1A monomer on the *Ap* DS (PDB: 1VS1). **d** Binding interaction model of divalent metal and Aro1A. The purple ball represents Mn^2+^; the blue stick represents the four binding residues; the black dotted lines represent inferred coordinating interactions

The optimal complexus crystal template of a DAHPS from *A. pernix* (Accession number: 1VS1.1.A) was selected for the homologous modeling of Aro1A on the basis of the SWISS-MODEL analysis. This template has the best Global Model Quality Estimate (0.77) and Quaternary Structure Quality Estimate (0.81) (Waterhouse et al. 2018). Figure 3a shows the tetramer of Aro1A that resulted from homology modeling. The monomeric structure of Aro1A is a (β/α)_8_ barrel structure (Fig. 3b), which was highly similar to that of 1VS1 (Fig. 3c). The results of homologous modeling showed a divalent metal ion (Mn^2+^) among four conserved metal binding residues (C36, H206, E232, and D243) (Fig. 3d).

**Fig. 4:**
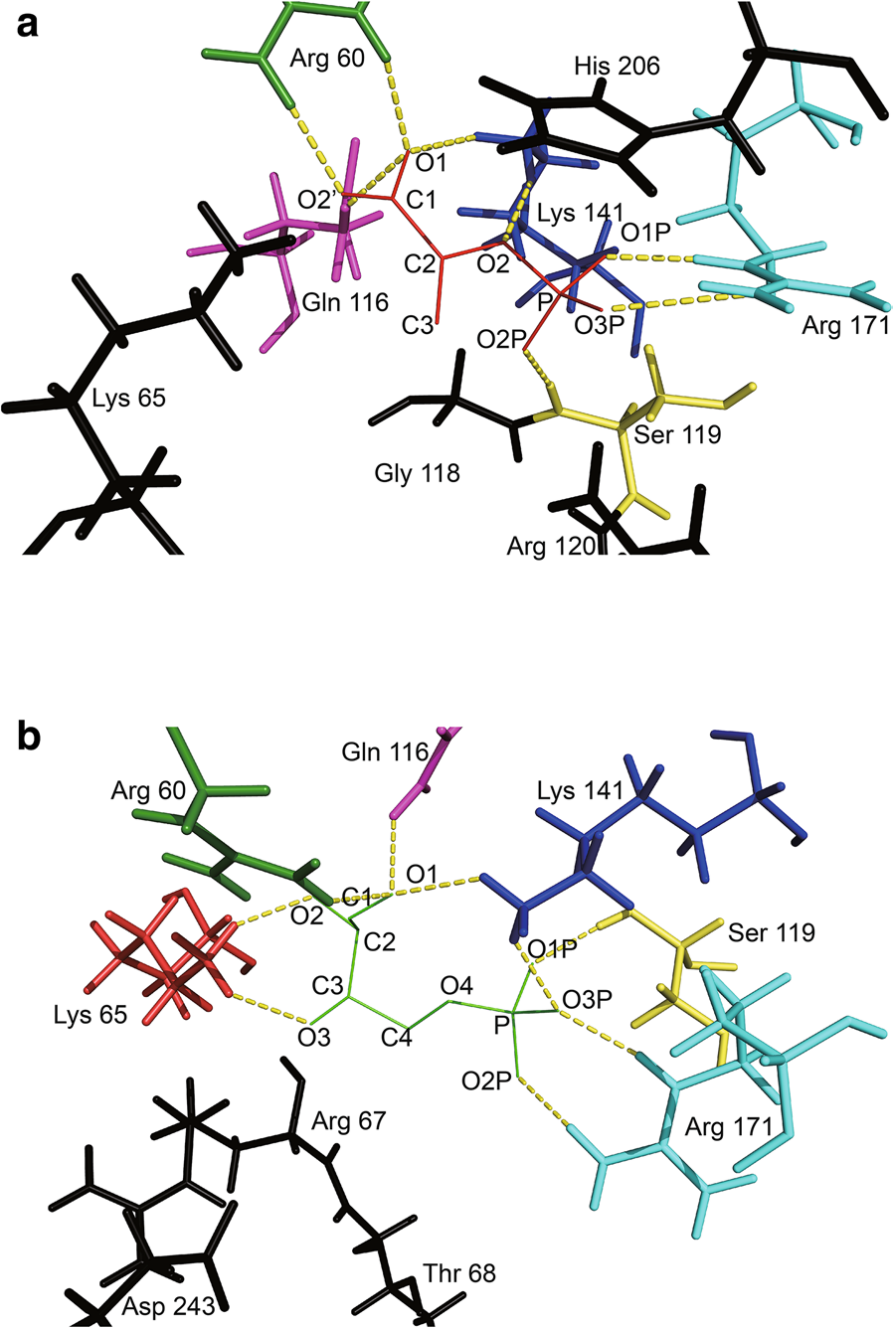
Docking model of Aro1A with substrates. **a** Docking model of PEP and Aro1A. **b** Docking model of E4P and Aro1A. PEP is shown as the red line, E4P is shown as the green line. The atomic names of these two substrates are labeled. The yellow dotted lines represent the hydrogen-bonding interactions. Residues are drawn as stick: Arg 60 (green), Lys 65 (red), Gln 116 (magenta), Ser 119 (yellow), Lys 141 (blue), and Arg 171 (cyan). Black sticks are the residues predicted to be associated with substrate binding in the alignment but not in docking

The results of molecular docking analysis showed that PEP combined with five residues (R60, Q116, S119, K141, and R171) through eight hydrogen bonds (Fig. 4a). Furthermore, E4P combined with six residues (R60, K65, Q116, S119, K141, and R171) through nine hydrogen bonds (Fig. 4b).

### Expression and purification of Aro1A in *E. coli*

**Fig. 5:**
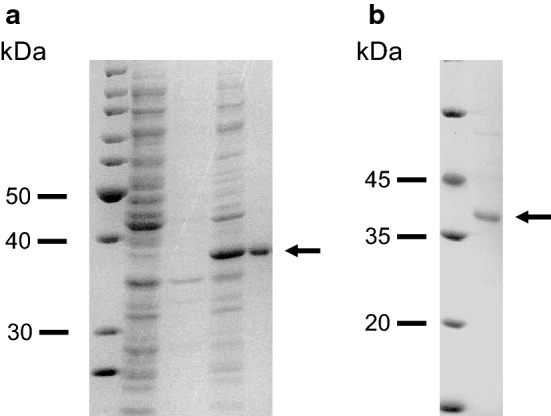
Analysis of expression and purification of Aro1A through SDS–PAGE. **a** Lane 1, molecular mass standards; lane 2, lysate supernatant of empty vector-carried expression strain; lane 3, lysate precipitate of empty vector-carried expression strain; lane 4, lysate supernatant of Aro1A-expressed strain; and Lane 5, lysate precipitate of Aro1A-expressed clone. **b** Lane 1, molecular mass standards; and Lane 2, purified Aro1A

Plasmid pET30a(+) with *aro1A* was transformed into competent cells of *E. coli* Rosetta (DE3). The transformed cells were cultivated by introducing IPTG. Cell extracts expressing Aro1A were subjected to SDS–PAGE. The results of SDS–PAGE indicated that cell lysate contained the target protein with a size of approximately 37 kDa (Fig. 5a). The protein was consistent with the predicted molecular weight. Furthermore, Fig. 5a shows that the quantity of soluble protein was more than 80%. The recombinant Aro1A protein was purified with Ni-IDA and analyzed via magnetic agarose chromatography (Fig. 5b).

### Effects of temperature, pH, and divalent metal ion on Aro1A

**Fig. 6:**
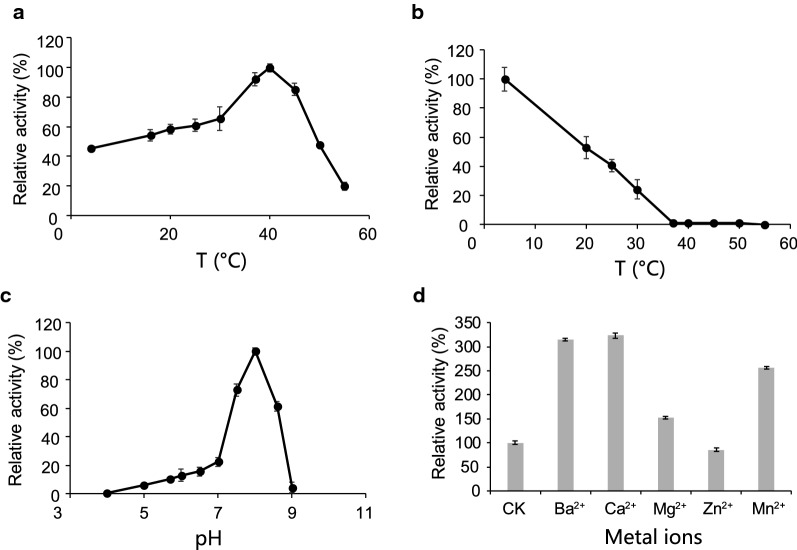
Effects of temperature, pH, and divalent metal ion on Aro1A. **a** Optimum reaction temperature of Aro1A, **b** thermostability of Aro1A, **c** optimum reaction pH of Aro1A, and **d** activation of Aro1A by divalent metal ions

Figure 6 shows the influence of temperature, pH, and divalent metal ions on the activity of Aro1A. The enzymatic activity of Aro1A was examined at different temperatures (4 °C, 16 °C, 20 °C, 25 °C, 30 °C, 37 °C, 40 °C, 45 °C, 50 °C, and 55 °C) and pH 6.8. Results showed that the optimal temperature was 40 °C. The enzymatic activity was more than 60% when the temperature was within 30 °C–47 °C (Fig. 6a). The thermostability of Aro1A was also tested. Furthermore, the enzymatic activity of Aro1A was analyzed under optimal reaction conditions after incubation at 4 °C, 20 °C, 25 °C, 30 °C, 37 °C, 40 °C, 45 °C, 50 °C, and 55 °C for 2 h, and the relative enzymatic activity at 4 °C was marked as 100%. Figure 6b shows that Aro1A had approximately 50% enzymatic activity at 20 °C. This activity greatly decreased to less than 5% when maintained at 37 °C–50 °C for 2 h. Furthermore, Aro1A lost its enzymatic activity when the temperature was increased to 55 °C.

The enzymatic activity of Aro1A at different pH levels (4.0, 5.0, 5.6, 6.0, 6.8, 7.0, 7.5, 8.0, 8.5 and 9.0) and 40 °C was also evaluated. Figure 6c shows that the optimal pH for Aro1A was 8.0, and the enzymatic activity was more than 60% when pH was within 7.3–8.5.

Ba^2+^, Ca^2+^, Mg^2+^, Zn^2+^, and Mn^2+^ were used in the enzymatic reaction system to determine the activation of Aro1A (Fig. 6d). Ba^2+^ and Ca^2+^ stimulated the activity of Aro1A to more than 300%, and Mn^2+^ stimulated such activity to more than 250%. Meanwhile, Mg^2+^ and Zn^2+^ had no substantial effect on the activity.

### Kinetic analysis

The enzymatic reaction rate was analyzed when the substrate concentration was 0.1–0.5 μM at optimal reaction conditions. The molecular kinetic parameters of Aro1A were examined via the Lineweaver–Burk double-reciprocal graphing method (Additional file 1: Fig. S4). The measured parameters were as follows: *K*_*m*_^PEP^ = 19.58 μM, *V*_*max*_^PEP^ = 29.02 μM·min^−1^, *k*_cat_^PEP^ value = 17.31 s^−1^, and *k*_cat_^PEP^/*K*_*m*_^PEP^ = 0.88 s^−1^ μM^−1^ (Table 2).

**Table 2:** Enzymatic property of representative DAHPSs that were expressed in the *E. coli* system

Organism	Protein	T	OpH	OT (°C)	*K*_*m*_^PEP^ (µM)	*k*_cat_^PEP^ (s^−1^)	*k*_cat_^PEP^/*K*_*m*_^PEP^ (s^−1^ µM^−1^)	References
*N. meningitidis*	*Nme* DS	I_α_	–	40	11	25	2.3	Cross et al. (2013)
Unculture Microorganisms	Aro1A	I_β_	8.0	40	19.58	17.31	0.88	This study
*Geobacillus sp.*	*Gsp* DS	I_β_	–	–	87	45	0.52	Nazmi et al. (2016)
*T. maritima*	*Tm* DS	I_β_	6.3	90	9.5	7.6	0.8	Wu et al. (2003)
*P. furiosus*	*Pfu* DS	I_β_	–	–	120	1.5	0.01	Schofield et al. (2004)
*B. subtilis*	*Bs* DS	I_β_	9.0	–	139	4.6	0.03	Wu et al. (2005)
*P. gingivalis*	*Pg* DS	I_β_	9.0	–	421	337	0.80	Wu and Woodard (2006)
*A. pernix*	*Ap* DS	I_β_	5.7	95	891	1.0	0.001	Zhou et al. (2012)
*M. tuberculosis*	*Mt* DS	II	–	–	37	3.1	0.08	Webby et al. (2005a)
*C. glutamicum*	*Cg* AroF	II	–	–	160	0.35	0.002	Liu et al. (2008)
	*Cg* AroG				8520	1.65	0.0002	

*T* DAHPS type, *OpH* optimal pH, *OT* optimal temperature

## Discussion

### Construction of the metagenomic library

The metagenomic DNA was directly extracted from the subtropical mangrove coastal wetland sediments. The constructed library contained a genome pool of the microorganisms in the wetland sediments, including that of uncultured microorganisms. Further analysis of randomly selected recombinant plasmids revealed that the foreign DNA fragments in pUC118 vector were highly diverse. This result also confirmed that the metagenomic library contained DNA molecules from uncharacterized genomes and that the metagenome of naturally occurring microbacteria contained an immense pool of genes; most of these genes could not be represented by pure and enrichment cultures established under certain selective conditions (Westmann et al. 2018). A new type I_β_ DAHPS gene (*aro1A*) was identified in a metagenomic library by using a sequence-based screening strategy from the subtropical mangrove sediment.

### Bioinformatics analysis of Aro1A protein

Relatively low consistence of sequence existed among DAHPSs; in particular, the sequence consistence between type I and type II is only 10% (Webby et al. 2005a). However, different DAHPSs have highly similar catalytic structural domain of the (β/α)_8_ barrel structure (König et al. 2004; Light et al. 2012; Nazmi et al. 2016; Shumilin et al. 1999, 2004; Sterritt et al. 2018; Webby et al. 2005a). The results of multiple sequence alignment reflected a similar situation. Only metal ion binding sites were totally conserved, and most of the DAHPSs had low sequence consistence. α-Helix and β chains of the catalytic structural domain shared a similar motif (Fig. 2).

The results of multiple sequence alignment revealed that the highly conserved residues in Aro1A involved in the combination of substrate binding sites and divalent metal ligands in other DAHPS enzymes (König et al. 2004; Wu and Woodard 2006) were completely conserved (Fig. 2). Four conserved binding residues (C36, H206, E232, and D243) were found with Mn^2+^ in the Aro1A protein (Fig. 3d). The motif 58VLRGGAFKPRT68 in Aro1A was highly conserved in type I_β_ DAHPS. R60 and K65 in this motif combined with PEP in *Mt* DS, *Tm* DS, *Pfu* DS, and *Ap* DS and had non-bonded contact with PEP in *Ec* AroG. In addition, R67 and T68 in the abovementioned motif were the binding sites of E4P in *Tm* DS and were predicted to be the binding sites of E4P in *Mt* DS, *Ec* AroG, and *Pfu* DS. This finding implied that 58VLRGGAFKPRT68 was the motif that participated in the PEP and E4P binding for Aro1A protein. The G118 in motif 118GSR120 was a highly conserved non-bond-contacting residue of PEP. The corresponding residue of S119 was Ala in *Ec* AroG, *Tm* DS, *Pfu* DS, and *Ap* DS and Glu in *Mt* DS. These residues were the binding sites of PEP, and R120 was the completely conserved binding site of PEP. Therefore, 118GSR120 also participated in the binding of PEP in Aro1A. H206 was another highly conserved residue that had non-bonded contact with PEP. This residue was the binding site of metal ligand and was annotated as a catalytic site in the analysis of other DAHPSs. The metal ligand binding site D243 bound with E4P in *Tm* DS and *Pfu* DS. All the above conserved residues, including K141 and R171, covered most of the ligand binding sites for proteins in multiple sequence alignments (Nazmi et al. 2014; Schofield et al. 2005; Shumilin et al. 1999, 2004; Webby et al. 2005a; Zhou et al. 2012). A previous research also indicated that the catalytic capacity of DAHPS was mainly based on the same (β/α)_8_ structure.

Six binding sites (R60, S119, K141, R171, K65, and R120) of PEP were found in the multiple sequence alignment based on the conservative property (Fig. 2). Based on the results of molecular docking analysis, R60, S119, K141, and R171 bound with PEP through a hydrogen bond. K65 bound with PEP in P*fu* DS, *Ec* AroG, and *Ap* DS. R120 bound with PEP in all five reference DAHPSs (Nazmi et al. 2014; Schofield et al. 2005; Shumilin et al. 1999, 2004; Webby et al. 2005a; Zhou et al. 2012). Furthermore, Q116 was predicted to bind with PEP in molecular docking. This residue was conserved among type I_β_ DAHPS in the multiple sequence alignment and bound with PEP in *Pfu* DS and *Ap* DS (Nazmi et al. 2014; Schofield et al. 2005; Zhou et al. 2012). The results of molecular docking analysis revealed that G118 and H206, which were conserved residues having non-bonded contact with PEP, were near PEP in Aro1A (Fig. 4a). This finding implied the importance of the two residues in PEP binding.

The predicted binding sites of E4P in the multiple sequence alignment were only R67, T68, and D243 residues, which were adjacent, but not bound, to E4P in molecular docking. In the molecular docking, the six residues bonded with E4P were R60, K65, Q116, S119, K141, and R171, which were nearly identical to the residues bonded with PEP. This result may be attributed to the similarity in the molecular structures of the two substrates. The two substrates were spatially closed in all reference protein structures. Furthermore, the binding mode of the five reference proteins with E4P in multiple sequence alignment is rarely researched. Among these proteins, only *Tm* DS with E4P was studied with crystal analysis of complexus (Shumilin et al. 2004). *Mt* DS and *Ec* DS were the binding sites of E4P based on the similarity of sulfate and phosphate groups (Shumilin et al. 1999, 2004; Webby et al. 2005a). The binding of E4P was not analyzed for *Pfu* DS and *Ap* DS (Nazmi et al. 2014; Schofield et al. 2005; Zhou et al. 2012). Although the binding mode of E4P and DAHPS is unclear, the possible binding sites were analyzed via molecular docking.

Based on the combined results of molecular docking and multiple sequence alignment, Aro1A was similar with other DAHPSs because they all had a “conserved” ligand-binding space to accommodate a divalent metal ion (PEP and E4P). The space included, but not limited to, totally conserved residues and a motif. We speculated that Aro1A was similar with *Pfu* DS and *Ap*DS based on the following: (1) only a catalytic part composed of (β/α)_8_ barrel existed, and (2) no part for the regulation on the N terminus or C terminus, indicating that Aro1A was not inhibited by the feedback of downstream aromatic amino acid.

### Enzymatic property of Aro1A

The optimal temperature (40 °C) of Aro1A was close to that of the DAHPS from *N. meningitis* (Table 2). The temperature activity was similar to that of *N. meningitis* (Cross et al. 2013). The sequence length, the amino acid composition of key motif, and the secondary structural arrangement of Aro1A (Fig. 2) were almost consistent with those of DAHPS from *A. pernix* and *P. furiosus*. The similarity of Aro1A and these two DAHPS was around 50%. In addition, Aro1A and the DAHPS from *T. maritima* (ACCESSION: Q9WYH8.1) had the highest similarity. However, the optimal temperatures of the DAHPS from *A. pernix* and *T. maritima* were 95 and 90 °C, respectively (Table 2). These enzymes have good thermostability (at least 60 °C) (Schofield et al. 2004; Wu et al. 2003; Zhou et al. 2012). Hence, although Aro1A and these DAHPS from thermophiles had highly similar sequence and structure, they apparently had different optimal temperature and thermostability. The difference among these DAHPS in temperature response requires further evaluation.

The optimal pH of Aro1A was 8.0. This pH was higher than that of the acidic DAHPs from *Tm* DS and *Ap* DS and was similar with that of DAHPS from *Bacillus subtilis* and *Porphyromonas gingivalis* (Table 2).

To date, all the reported DAHPSs are metalloenzymes (Wu et al. 2005) that can be activated by a series of divalent metal ions. However, the activation mechanism of different metal ions considerably varies for different DAHPSs. Similar with the Aro1A protein, DAHPSs from *C. glutamicum, P. furiosus*, *T. maritima, Actinosynnema, M. tuberculosis, H. pylori, Pseudomonas aeruginosa*, and *N. meningitidis* (Cross et al. 2013; Liu et al. 2008; Ma et al. 2012; Schofield et al. 2004; Sterritt et al. 2018; Webby et al. 2005a, b; Wu et al. 2003) can be stimulated with Mn^2+^ ion. Ba^2+^ and Ca^2+^ had no effect for the DAHPS from *N. meningitis* (Cross et al. 2013). However, Ba^2+^ and Ca^2+^ can stimulate the activity of Aro1A protein. Furthermore, Mg^2+^ had relatively weak activation action on Aro1A but had better effect on DAHPS from *C. glutamicum* and *Actinosynnema* (Liu et al. 2008; Ma et al. 2012). Similar results can be found when the activation capacities of metal ions are compared.

The catalytic capacity of Aro1A was higher than that of the other type I_β_ DAHPSs (Table 1), and Aro1A had relatively moderate optimal temperature (Table 2). Furthermore, the enzyme was an inherent DAHPS without feedback inhibition structure. Hence, Aro1A can be potentially used in the industrial production of aromatic amino acids, provided that the thermostability was solved by molecular modification.

## Additional file

**Additional file 1.** Additional figures.

## Abbreviations

DAHP: 3-deoxy-d-arabino-heptulosonate-7-phosphate
DAHPS: 3-deoxy-d-arabino-heptulosonate-7-phosphate synthase
PEP: phosphoenolpyruvate
E4P: d-erythrose 4-phosphate
IPTG: isopropyl β-d-1-thiogalactopyranoside
PCR: polymerase chain reaction
