# Supplementary material for: Isolation and biochemical characterization of a metagenome-derived 3-deoxy-d-arabino-heptulosonate-7-phosphate synthase gene from subtropical marine mangrove wetland sediments
Source: AMB Express. 2019 Feb 4;9:19. doi: 10.1186/s13568-019-0742-4 (PMC6362186; doi:10.1186/s13568-019-0742-4)
Supplement: Supplementary file 1 — Additional file 1. Additional figures. [file 13568_2019_742_MOESM1_ESM.docx]

**Additional Materials**

**Isolation and Biochemical Characterization of a Metagenome-derived 3-Deoxy-D-arabino-heptulosonate-7-phosphate Synthase Gene from Subtropical Marine Mangrove Wetland Sediments**

**Huaxian Zhao^1, †^, Hua Gao^1, †^, Kai Ji^1^, Bing Yan^2^, Quanwen Li^1^, Shuming Mo^1^, Minggang Zheng^4^, Qian Ou^1^, Bo Wu^1^, Nan Li^3^** ***, and Chengjian Jiang^1, 2**^**

^1^ State Key Laboratory for Conservation and Utilization of Subtropical Agro-bioresources, College of Life Science and Technology, Guangxi University, 100 Daxue East Road, Nanning, Guangxi, 530004, PR China.

^2^ Guangxi Key Laboratory of Mangrove Conservation and Utilization, Guangxi Mangrove Research Center, Guangxi Academy of Sciences, 92 Changqing Road, Beihai, Guangxi, 536000, PR China.

^3^ Key Laboratory of Environment Change and Resources Use in Beibu Gulf (Guangxi Teachers Education University), Ministry of Education, 175 Mingxiu East Road, Nanning, Guangxi, 530001, PR China.

^4^ The First Institute of Oceanography, State Oceanic Administration of China, 6 XianXiaLing Road, Qingdao 266061, PR China.

**^†^** These authors contributed equally to this work.

* and ** : Corresponding Author:

*** Nan Li:**

Tel: +86-771-3239403; Fax: +86-771-3237873

nli@yic.ac.cn

**** Chengjian Jiang:**

Tel: +86-771-3239403; Fax: +86-771-3237873

[jiangcj0520@gmail.com](mailto:jiangcj0520@gmail.com)


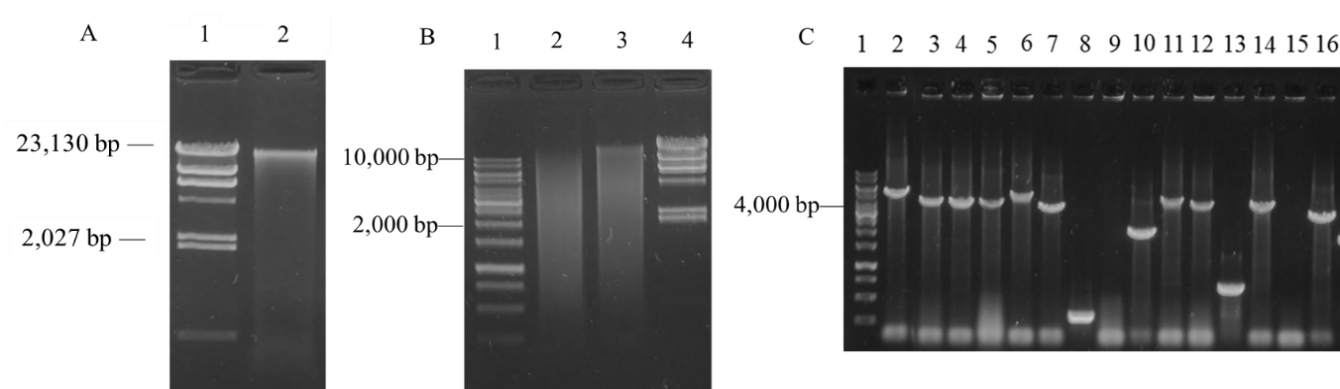


**Additional file 1: Fig. S1.** Construction of metagenomic library. (A) Lane 1, DNA marker; Lane 2, metagenome DNA. (B) Lanes 1 and 4, DNA marker; Lane 2, *Hinc* II-digested metagenome DNA; Lane 3, *Sma* I-digested metagenome DNA. (C) Lane 1, DNA marker; Lanes 2–16, PCR detection of the inserted DNA.


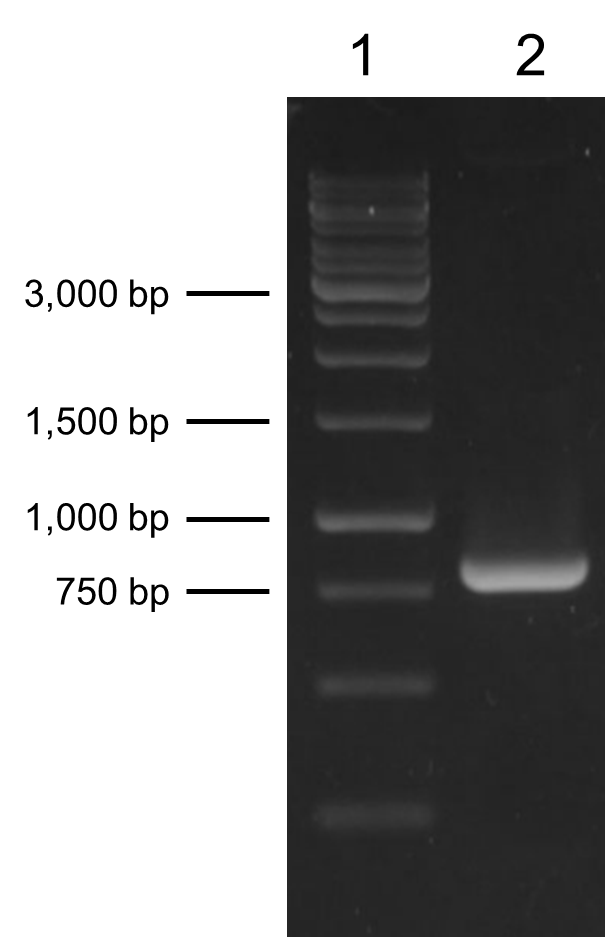


**Additional file 1: Fig. S2.** PCR production of *aro1A*. Lane 1, DNA marker; Lane 2, PCR production of *aro1A*.


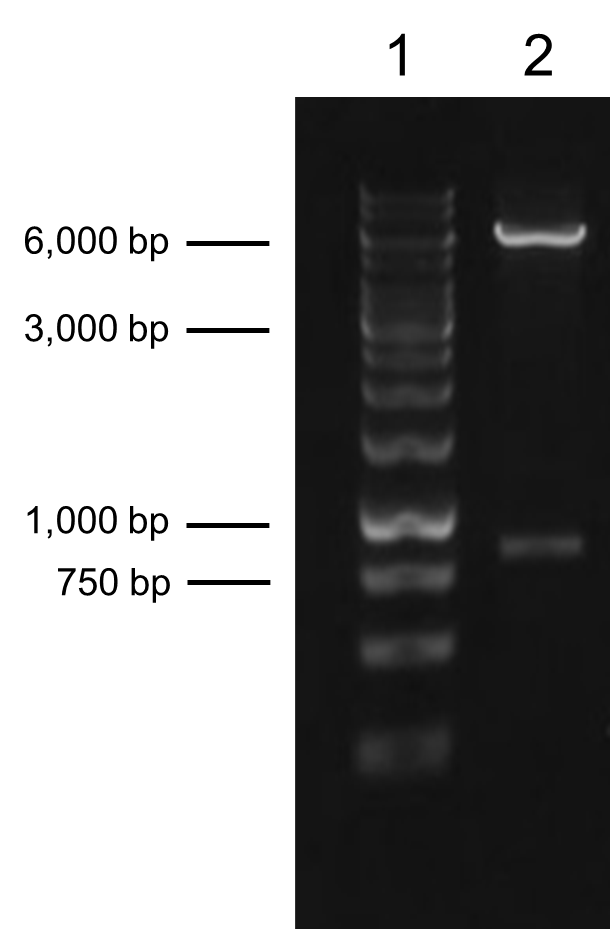


**Additional file 1: Fig. S3.** Identification of recombinant expression plasmids via double digestion with *Hin*d III and *Xho* I. Lane 1, DNA marker; Lane 2, recombinant plasmid pET30a (+)*-aro1A* digested by *Hin*d III and *Xho* I.


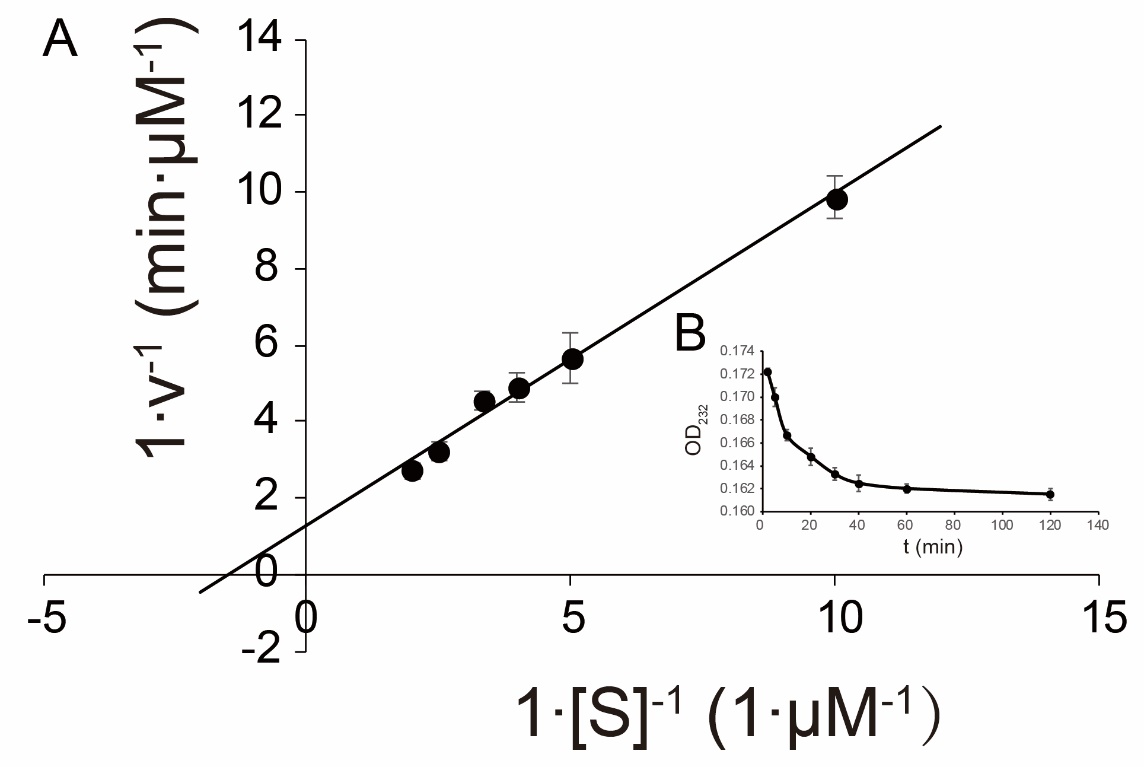


**Additional file 1: Fig. S4.** Determination of kinetic parameters of Aro1A. (A) Lineweaver–Burk double-reciprocal graph. (B) Reaction progress curve.
